# Thermal nociception as a measure of non-steroidal anti-inflammatory drug effectiveness in broiler chickens with articular pain^☆^

**DOI:** 10.1016/j.tvjl.2013.09.013

**Published:** 2013-12

**Authors:** Gina Caplen, Laurence Baker, Becky Hothersall, Dorothy E.F. McKeegan, Victoria Sandilands, Nick H.C. Sparks, Avril E. Waterman-Pearson, Joanna C. Murrell

**Affiliations:** a School of Veterinary Sciences, University of Bristol, Langford, Bristol, UK; b Avian Science Research Centre, Scottish Agricultural College, Auchincruive, Ayr, UK; c Institute of Biodiversity, Animal Health and Comparative Medicine, University of Glasgow, Glasgow, UK

**Keywords:** Broiler, Lameness, Nociceptive, NSAID, Pain

## Abstract

Pain associated with poultry lameness is poorly understood. The anti-nociceptive properties of two non-steroidal anti-inflammatory drugs (NSAIDs) were evaluated using threshold testing in combination with an acute inflammatory arthropathy model. Broilers were tested in six groups (*n* = 8 per group). Each group underwent a treatment (saline, meloxicam (3 or 5 mg/kg) or carprofen (15 or 25 mg/kg)) and a procedure (Induced (arthropathy-induction) or sham (sham-handling)) prior to testing. Induced groups had Freund’s complete adjuvant injected intra-articularly into the left intertarsal joint (hock). A ramped thermal stimulus (1 °C/s) was applied to the skin of the left metatarsal. Data were analysed using random-intercept multi-level models.

Saline-induced birds had a significantly higher skin temperature (± SD) than saline-sham birds (37.6 ± 0.8 °C vs. 36.5 ± 0.5 °C; *Z* = −3.47, *P* < 0.001), consistent with an inflammatory response. Saline was associated with significantly lower thermal thresholds (TT) than analgesic treatment (meloxicam: *Z* = 2.72, *P* = 0.007; carprofen: *Z* = 2.58, *P* = 0.010) in induced birds. Saline-induced birds also had significantly lower TT than saline-sham birds (*Z* = −2.17, *P* = 0.030). This study found direct evidence of an association between inflammatory arthropathies and thermal hyperalgesia, and showed that NSAID treatment maintained baseline thermal sensitivity (via anti-nociception). Quantification of nociceptive responsiveness in a predictable broiler pain model identified thermal anti-hyperalgesic properties of two NSAIDs, which suggested that therapeutically effective treatment was provided at the doses administered. Such validation of analgesic strategies will increase the understanding of pain associated with specific natural broiler lameness types.

## Introduction

Lameness has been reported to affect almost 30% of intensively-reared broilers in the UK (Knowles et al., 2008). The cause can be infectious or non-infectious, and may involve tendons, joints, ligaments and bones (Bradshaw et al., 2002). Immobility has obvious welfare implications if birds are unable to access drinkers and feeders, but the prevalence and severity of pain associated with lameness in poultry is poorly understood. Joint inflammation in humans has been correlated to pain, especially during activity (Gregory, 2010), and inflammatory arthropathy has been identified within lame broiler hock joints (Corr et al., 2003). Pain assessment in non-human species is complicated by an inability to communicate verbally and must therefore be inferred using indirect measures.

Non-steroidal anti-inflammatory drugs (NSAIDs) are routinely used to manage pain associated with osteoarthritis in dogs and cats (Papich, 2008) and may have therapeutic potential in poultry. Although there is some evidence for NSAID treatment improving lame broiler walking ability, the heterogeneous nature of the studies (dose, route of administration, pathology, and end-measures) necessitates substantiation. Carprofen has been observed to improve lame broiler mobility (McGeown et al., 1999) and lame broilers were found to preferentially select carprofen-spiked feed in a self-selection experiment (Danbury et al., 2000). However, the latter study was subject to variations in feed consumption (inter-bird) and lameness severity (intra-bird) and lame birds failed to demonstrate higher carprofen plasma concentrations than non-lame birds at the end of the trial. Although pharmacodynamic and behavioural data are currently lacking, meloxicam also has therapeutic potential for use in broilers, demonstrating a promisingly high bioavailability in poultry following intravenous administration (Baert and De Backer, 2002, 2003).

An arthritis model has been used to determine optimum analgesic dosing rates (including carprofen) via the identification of (pain-related) behavioural changes in laying hens (Hocking et al., 1997, 2001, 2005). However, a similar study would be unsuitable for meat-type chickens since ‘resting’, the key behaviour that decreased in layers following analgesic treatment, is a baseline broiler behaviour; sound broilers spend approximately 80% of their time sitting (Weeks et al., 2000). In addition, it is possible that chicken behaviour may not accurately reflect a pain state since prey species are less likely to display overt pain-associated behaviour that may increase predation risk (Livingston, 1994). Quantitative sensory testing offers an alternative means to evaluate analgesic drug efficacy in broilers. Application of standardised noxious stimuli (duration and intensity) allows the functional state of the nociceptive system to be investigated and the anti-nociceptive properties of drugs to be quantified. Measurement of thermal nociceptive threshold has been widely used to establish the anti-nociceptive efficacy of drugs in a range of species including rats (Nagakura et al., 2003), cats (Taylor et al., 2007), fish (Nordgreen et al., 2009), and an early study on fowl (Hughes, 1990). An elevated thermal threshold indicates an anti-nociceptive action and can be used as a biomarker of post-therapeutic effect and duration.

Due to the anti-hyperalgesic, rather than analgesic, mechanism of action of NSAIDs, thermal nociceptive threshold is useful to measure NSAID efficacy when administered immediately prior to, or after induction of inflammation rather than in naïve animals (Taylor et al., 2007; Giraudel et al., 2009; Jeunesse et al., 2011). Slowly adapting mechanoreceptors are present within the skin of the chicken tarso-metatarsus (Gentle et al., 2001) and these are sensitised following induced inflammation (Gentle and Thorp, 1994; Gentle, 1997). We have recently reported baseline thermal nociceptive threshold data for broilers (Hothersall et al., 2011) and now predict that the application of this robust and validated methodology, to a predictable pain model, will allow us to define effective poultry-relevant analgesic therapy.

## Materials and methods

### Birds and husbandry

A group (*n* = 90) of 17-day old, male Ross 308, broiler chickens was obtained from a commercial flock, 3 days prior to initial data collection, and transferred to the research facility (Scottish Agricultural College, Auchincruive). All birds were non-lame, having been assessed on farm as ‘gait score (GS) 1’ (Kestin et al., 1992). Upon arrival each broiler was identified with a numbered wing tag (Roxan Developments) and then housed in four groups of 20 (with 10 birds as spares). Birds were maintained on wood shavings at an ambient temperature of 21 °C, at 60–70% humidity, and on a lighting regime of 18 h light and 6 h dark in pens that exceeded the UK legal space requirements for the housing of broilers undergoing experimental procedures. Food (standard grower pellets) and water were provided ad libitum. Birds were humanely euthanased at the end of the study.

### Procedure and treatment allocation

From the potential test cohort, 48 broilers, randomly allocated into four sub-groups (*n* = 12), were used for data collection. Test birds were weighed immediately prior to receiving experimental treatment. There was no difference in bodyweight (BW) between groups (1.00 ± 0.06 kg, mean ± SD). On each day of a 4-day experimental period all individuals within a single sub-group underwent both an analgesic treatment (minus 6 h) and an experimental procedure (minus 4 h), prior to thermal threshold testing. Analgesic treatment was administered via a 1 mL subcutaneous injection to the back of the neck, as follows: (1) saline control; (2) 3 mg/kg meloxicam (*M*_low_; 20 mg/mL Metacam injectable solution, Boehringer Ingelheim); (3) 5 mg/kg meloxicam (*M*_high_); (4) 15 mg/kg carprofen (*C*_low_; 50 mg/mL Rimadyl injectable solution, Pfizer Animal Health), and (5) 25 mg/kg carprofen (*C*_high_).

Because this study involved a species for which the effectiveness of other common analgesics is not yet well established, the use of a positive control group in place of a negative control group, although recommended, was not feasible in the evaluation of the analgesic effects of meloxicam and carprofen. At no point during this study was any bird seen to become more than ‘moderately lame’ (defined as exceeding GS 3).

Experimental procedures consisted of either (1) acute arthropathy induction (Induced), achieved via intra-articular injection (using a 22 G needle) of 0.4 mL Freund’s complete adjuvant (FCA), an immunopotentiator, into the left intertarsal joint (hock), or (2) sham handling with no injection (Sham). One millilitre of FCA contained 1 mg heat killed and dried *Mycobacterium tuberculosis*, 0.85 mL paraffin oil and 0.15 mL mannide monooleate. Within each sub-group the procedure and treatment were allocated as follows: Saline-induced (*n* = 2), Saline-sham (*n* = 2), *M*_low_-induced (*n* = 1), *M*_low_-sham (*n* = 1), *M*_high_-induced (*n* = 1), *M*_high_-sham (*n* = 1), *C*_low_-induced (*n* = 1), *C*_low_-sham (*n* = 1), *C*_high_-induced (*n* = 1), *C*_high_-sham (*n* = 1).

### Threshold testing

Threshold testing was performed by one investigator (GC) using equipment commissioned from Topcat Metrology (for full details of the equipment and testing procedure, see Hothersall et al., 2011) and conducted under Home Office licence (PPL 60/3711). To ensure complete visibility birds were transferred to solid floor wire cages (dimensions: 750 mm in length × 500 mm width × 850 mm height) prior to testing (*n* = 6 birds per cage).

A thermal probe was attached to the skin covering the lateral aspect of the tarsometatarsus (immediately below the left hock) and left to equilibrate to skin temperature (ST) for 15 min. Four consecutive thermal threshold (TT) measurements were then recorded, each separated by at least 10 min. To obtain each TT value, the starting ST was noted and then the probe was heated at a rate of 1 °C/s until a characteristic behavioural response was observed (e.g. stand from sitting, shuffle, or raised leg), or a pre-specified cut-out temperature was reached (50 °C). Heating of the probe was then immediately stopped and the TT measure (maximum probe temperature reached during heating) was recorded. The ‘excursion temperature’ (δT), the rise in temperature above ST required to produce a response, was calculated by subtracting each ST from the respective TT value. Skin temperature returned to baseline (i.e. that recorded at the end of the equilibration period prior to initialising testing) between each of the four threshold tests.

Although the investigator (GC) was unaware of test treatment allocation (saline or NSAID) and NSAID dose (high or low), nor administered the experimental procedure (sham-handling or induced-arthropathy), it was difficult to maintain absolute blinding due to the obvious lameness and swelling in birds that received FCA.

### Data analysis

Multilevel modelling software (MLwiN v2.22) was used to create random-intercept nested models reflecting the hierarchical structure of the data set; the measure of interest was selected as the response variable, and the nested hierarchy comprised two levels (test number and bird ID). Standardised residuals were plotted against normal scores for all models and any obvious outliers were omitted from the analysis. The significance of individual predictors in a model was tested using *Z*-tests, whereby the coefficient was divided by the standard error of coefficient to generate respective *Z*-values. *P*-values were calculated as the area of the Normal distribution greater than or equal to the *Z*-value, multiplied by two (two-tailed analysis). Likelihood ratio tests were employed to investigate whether an interaction between Procedure and Treatment was significant, the likelihood ratio test statistic (LR) being the difference between the 2 × log likelihood values for the null and alternative models. Since an initial analysis of the data set found no dose effect for either analgesic on either threshold measure (TT or δT), it was decided to combine doses within analgesic type (Treatment). *P* values ⩽0.05 were considered to be statistically significant.

## Results

Values for the three thermal threshold measures are provided within Table 1.

### Skin temperature (ST)

The procedure was associated with significantly different ST-values when treatment was accounted for (*Z* = 4.06, *P* < 0.001). Both NSAIDs were associated with significantly lower ST-values than saline (meloxicam: *Z* = −2.39, *P* = 0.016; carprofen: −3.12, *P* = 0.002), yet were themselves comparable (*Z* = 0.25, *P* = ns) (Table 1). There was no significant interaction between procedure and treatment (LR = 0.503, d.f. = 3, *P* = ns). Induced birds receiving meloxicam had comparable STs to those administered saline (*Z* = 1.44, *P* = ns) or carprofen (*Z* = −1.44, *P* = ns); however, carprofen treatment was associated with a lower STs than saline (*Z* = −2.27, *P* = 0.023). Saline-induced birds had higher STs than the saline-sham cohort (*Z* = −3.47, *P* < 0.001). A similar effect of procedure (higher ST in induced birds) was seen with meloxicam (*Z* = −3.56, *P* < 0.001), but not for carprofen (*Z* = −1.44, *P* = ns).

### Thermal threshold (TT)

**Fig. 1:**
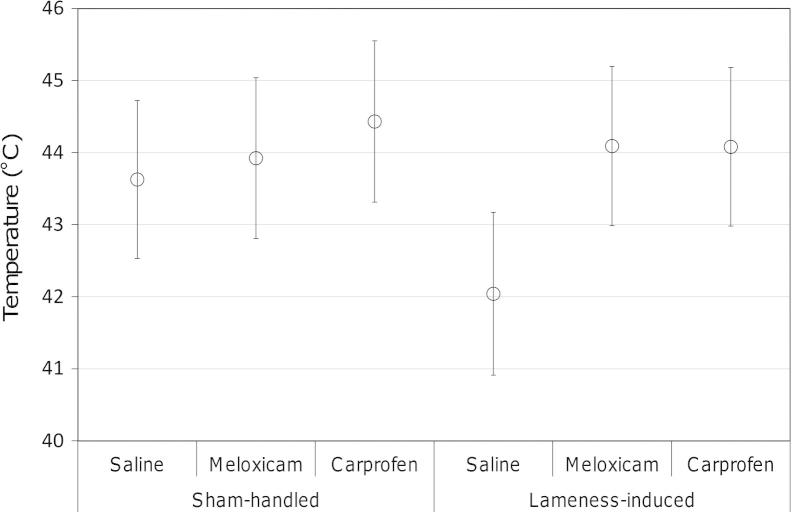
Thermal threshold values (±95% confidence) generated using a multilevel model to account for skin temperature. Data were recorded from broilers (*n* = 8, per group) in a repeated-measures study design following administration of a procedure (sham-handled or arthropathy-induced) and a treatment (saline, meloxicam or carprofen).

As ST was influenced by both procedure and treatment, it was deemed necessary to include ST as an additional covariate in models for the analysis of thermal threshold (TT). When both ST and treatment were accounted for, a comprehensive analysis of the data set revealed that procedure did not have a significant effect upon TT (*Z* = 1.34, *P* = ns). Overall, when both Sham and Induced birds were considered together, both NSAIDs were associated with significantly higher TT-values than saline (meloxicam: *Z* = 2.02, *P* = 0.043; carprofen: 2.43, *P* = 0.015), and no significant difference was evident between the two analgesics (*Z* = 0.44, *P* = ns). Although a significant interaction between procedure and treatment was not apparent (LR = 2.509, d.f. = 2, *P* = ns), the plotted model (±95% confidence intervals, Fig. 1) indicated that TT was lower in the Saline-Induced birds.

Within the Sham group TT-values were unaffected by treatment (saline vs. meloxicam: *Z* = 0.40, *P* = ns; saline vs. carprofen: *Z* = 0.94, *P* = ns; meloxicam vs. carprofen: *Z* = 0.55, *P* = ns). In the Induced group, saline was associated with significantly lower TT-values than either of the analgesic treatments (meloxicam: *Z* = 2.72, *P* = 0.007; carprofen: *Z* = 2.58, *P* = 0.010); and TT-values for the two analgesics were comparable (*Z* = 0.08, *P* = ns, Fig. 1). Procedure had a significant effect upon TT with saline (*Z* = 2.17, *P* = 0.030); however, no difference was evident with analgesic (meloxicam: *Z* = 0.54, *P* = ns; carprofen: *Z* = 0.75, *P* = ns, Fig. 1).

In summary, the FCA model caused a decrease in thermal nociceptive threshold (as determined by TT), providing evidence of primary thermal hyperalgesia, and this effect was obtunded by the administration of either NSAID at the doses used.

## Discussion

The mean leg skin temperature (ST) of 36.5 ± 0.5 °C recorded in the control (Saline-sham) birds was towards the top end of the range previously reported (35.8 ± 1.2 °C; (Hothersall et al., 2011). This may have been due to age-related metabolic differences, since the birds in the current study were at least 1 week younger than those used previously (Buyse et al., 1994). The mean thermal nociceptive threshold (TT) of 43.7 ± 1.8 °C was also within the range documented by the validation study, as was the mean excursion temperature (δT) of 7.2 ± 2.1 °C (TT: 42.5 ± 2.5 °C; δT: 6.7 ± 2.8 °C; Hothersall et al., 2011). The range of threshold values recorded (37.0–48.4 °C) was measured using a relatively slow rate of heating (1.0 °C/s), indicating that the thermal stimulus activated polymodal C-fibre nociceptors (range: 39–61 °C), present within the skin covering the tarso-metatarsus (Gentle et al., 2001). Since slow (0.9 °C/s) and fast (6.5 °C/s) rates of skin heating have been preferentially found to activate either C- or Aδ-thermal nociceptors, respectively, in rats (Yeomans and Proudfit, 1996) the rate of heating employed within the current study would have been too slow to stimulate Aδ-fibres.

The intra-articular administration of FCA following saline and meloxicam treatment significantly increased skin temperature in the vicinity of the test site (consistent with visible hock swelling), compared to sham-handling. As basal systemic temperature readings were not measured we cannot say whether the immunopotentiator produced localised cutaneous hyperthermia or a systemic febrile response. Meloxicam and carprofen have antipyretic properties and reduce body temperature in febrile states. The test doses were selected on the basis of previous avian studies (Hocking et al., 2005; Cole et al., 2009; Paul-Murphy et al., 2009). Since carprofen maintained peripheral skin temperature at a level comparable to the Sham group this suggests that, at the doses administered, it had a greater physiological efficacy than meloxicam.

As expected, treatment of the Sham group with NSAIDs did not affect TT-values and provided no evidence for reduced thermal sensitivity in these non-lame birds. The significantly lower TT-values recorded in the Saline-induced birds, compared to the Saline-sham cohort, indicated that experimentally induced arthropathy was associated with primary thermal hyperalgesia. Since thermal threshold was only measured near to the test site, and not in combination with a distant reference site (or using apparatus capable of stimulating Aδ-nociceptors), we were unable to determine whether the FCA model induced secondary hyperalgesia in addition to localised sensitisation. A FCA inflammatory model using the unilateral induction of inflammation near/within tibio-tarsal joints in rodents consistently failed to modify thermal thresholds in the contra-lateral paw (Chillingworth and Donaldson, 2003; Nordgreen et al., 2009). This would suggest that the broilers subjected to the FCA-procedure experienced increased noxious input from the inflamed joint and that the lower thresholds observed in response to the acutely applied thermal stimulus were due to a peripheral hypersensitivity of inflamed tissue, consistent with a state of acute inflammatory pain. Since the Induced birds given saline exhibited significantly reduced TT-values to those given analgesic, and there was no procedural-effect upon TT-values for either analgesic treatment, we can infer that both NSAID treatments were anti-hyperalgesic, restoring the thermal sensitivity of the Induced birds to ‘baseline’ and, as such, were therapeutically effective at the doses administered.

In preliminary studies we had expected to demonstrate primary thermal hyperalgesia in broilers with natural lameness, a feature which could subsequently be used to investigate the therapeutic potential of NSAID treatment. However, due to the complexities associated with linking lameness severity with pathology (Sandilands et al., 2011) we experienced difficulties in standardising our test cohorts (pathology was assigned retrospectively at post mortem), and this created problems with data interpretation for finalising appropriate dose (Hothersall et al., 2012). The current study is important as a primary proof-of-principle. NSAID treatments identified as suitable for reversing induced-hyperalgesia in birds with experimentally induced arthropathy can now be transferred to wider broiler lameness studies, to target naturally occurring inflammatory pathologies for experimental purposes.

## Conclusions

An acute model of articular pain was used in broiler chickens to provide direct evidence that carprofen and meloxicam maintained baseline threshold thermal anti-hyperalgesia for 6 h following subcutaneous administration. The quantification of nociceptive responsiveness using a predictable pain model will be useful to predict the efficacy of therapeutic treatments used treat naturally-occurring lameness in chickens.

## Conflict of interest statement

None of the authors of this paper has a financial or personal relationship with other people or organisations that could inappropriately influence or bias the content of the paper.

## Figures and Tables

**Table 1:** Thermal threshold data (°C: mean^a^ ± SD) recorded from broilers (*n* = 8, per group) following administration of a procedure (sham-handled or arthropathy-induced) and a treatment (saline, meloxicam or carprofen).

Procedure	Treatment	Skin temperature, ST	Thermal threshold, TT	Excursion temperature, δT
Sham	Saline	36.5 ± 0.5	43.7 ± 1.8	7.15 ± 2.09
	Meloxicam	35.4 ± 0.7	43.5 ± 1.5	8.09 ± 1.85
	Carprofen	35.4 ± 1.8	43.8 ± 2.1	8.41 ± 2.56

Induced	Saline	37.6 ± 0.8	42.7 ± 1.3	5.15 ± 1.09
	Meloxicam	37.0 ± 1.0	44.3 ± 1.5	7.39 ± 1.71
	Carprofen	36.5 ± 1.1	44.1 ± 1.6	7.65 ± 1.41

a Calculated using individual mean values averaged over four thermal threshold tests.
